# Conjugated Human Serum Albumin/Gold-Silica Nanoparticles as Multifunctional Carrier of a Chemotherapeutic Drug

**DOI:** 10.3390/ijms252413701

**Published:** 2024-12-21

**Authors:** Elena Morrone, Lucie Sancey, Fabien Dalonneau, Loredana Ricciardi, Massimo La Deda

**Affiliations:** 1 Department of Chemistry, Biology and Biotechnology, University of Perugia, 06123 Perugia, Italy; elena.morrone@dottorandi.unipg.it; 2 Department of Chemistry and Chemical Technologies, University of Calabria, 87036 Rende, Italy; 3 CNR-NANOTEC Institute of Nanotechnology, National Research Council, 87036 Rende, Italy; 4 Université Grenoble Alpes, INSERM U1209, CNRS UMR 5309, Institute for Advanced Biosciences (IAB), 38000 Grenoble, France; lucie.sancey@univ-grenoble-alpes.fr (L.S.); fabien.dalonneau@univ-grenoble-alpes.fr (F.D.)

**Keywords:** multifunctional nanoplatforms, phototherapy, chemotherapy, human serum albumin, tumor targeting

## Abstract

We report the design and development of a novel multifunctional nanostructure, RB-AuSiO_2__HSA-DOX, where tri-modal cancer treatment strategies—photothermal therapy (PTT), photodynamic therapy (PDT), chemotherapy—luminescent properties and targeting are integrated into the same scaffold. It consists of a gold core with optical and thermo-plasmonic properties and is covered by a silica shell entrapping a well-known photosensitizer and luminophore, Rose Bengal (RB). The nanoparticle surface was decorated with Human Serum Albumin (HSA) through a covalent conjugation to confer its targeting abilities and as a carrier of Doxorubicin (DOX), one of the most effective anticancer drugs in clinical chemotherapy. The obtained nanostructure was fully characterized through transmission electron microscopy (TEM), dynamic light scattering (DLS) and UV-visible spectroscopy, with a homogeneous and spherical shape, an average diameter of about 60 nm and negative ζ-potential value Singlet oxygen generation and photothermal properties were explored under green light irradiation. The interaction between DOX-HSA anchored on the nanoplatform was investigated by fluorescence spectroscopy and compared to that of DOX-HSA, pointing out different accessibility of the drug molecules to the HSA binding sites, whether the protein is free or bound to the nanoparticle surface. To the best of our knowledge, there are no studies comparing a drug–HSA interaction with that of the same protein anchored to nanoparticles. Furthermore, the uptake of RB-AuSiO_2__HSA-DOX into MDA-MB-231 mammary cells was assessed by confocal imaging, highlighting—at early time of incubation and as demonstrated by the increased DOX luminescence displayed within cells—a better internalization of the carried anticancer drug compared to the free one, making the obtained nanostructure a suitable and promising platform for an anticancer multimodal approach.

## 1. Introduction

Cancer is the second leading cause of death worldwide, with nearly 10 million deaths in 2020 [1]. Although technological advances have led to significant improvements in conventional cancer treatments, the development of a non-invasive, low-toxic and resolutive therapy remains an ongoing challenge.

Currently, an emerging strategy in clinical oncology is represented by a combinatorial therapeutic approach, which employs two or more treatment modalities to obtain a synergistic action with consequent superadditive (namely “1 + 1 > 2”) therapeutic effects [2].

In this field, colloidal nanomaterials have attracted a great deal of attention, proving to be an ideal scaffold that integrates multiple diagnostic-therapeutic functions onto a single platform [2,3]. Furthermore, due to their size, they are able to passively accumulate at the tumor site via the enhanced permeability and retention (EPR) effect [4], and their surface can be ad hoc-engineered and functionalized with active targeting moieties—such as proteins, aptamers, antibodies, etc.—to get a tumor-specific targeting [2,5,6].

Among them, gold-based nanoparticles have been shown to be promising agents for cancer therapy [7]. In particular, due to their photothermal conversion ability, they have been explored in photothermal therapy (PTT) [8,9], acting—upon irradiation at their plasmon resonance wavelengths or receiving radiant energy from antenna systems—as nano sources of localized heat, thereby inducing thermal tumor ablation [10]. Moreover, the generated thermal effects can also be exploited to trigger drug payload release with high spatial and temporal resolution [8,11].

Besides PTT, another well-known light-mediated cancer treatment is photodynamic therapy (PDT) [12]. PDT uses photosensitizing molecules able to absorb light of appropriate wavelengths and transfer the absorbed energy to molecular oxygen, generating singlet oxygen and/or—through electron transfer processes—other reactive oxygen species (ROS), including superoxide anion, hydrogen peroxide and hydroxyl radical, resulting in oxidative damage, and ultimately leading to tumor cell death, tumor-related vascular injury and activation of anti-tumor immunity [13,14,15].

Due to their unique advantages, such as non-invasiveness and low toxicity, both phototherapies, PTT and PDT, are becoming a revolutionary approach in combinatorial cancer treatment strategies in association with surgery, chemo, radio and immunotherapy [12,16,17,18], and in recent years, an ever-growing number of multifunctional nanoplatforms based on these therapeutic approaches have been reported [19,20,21,22]. In particular, Fan et al. reported a comprehensive review of the use of nanotechnology to achieve the “mission” of multimodal synergistic cancer therapy [2].

Human Serum Albumin (HSA) is the most abundant protein in the plasma, which plays an important physiological role in maintaining oncotic pressure and transporting a huge variety of both endogenous and exogenous compounds, such as hormones, long-chain fatty acids, metal ions and therapeutic agents [23]. Due to its intrinsic properties, such as biocompatibility, biodegradability, and low immunogenicity, it is widely used as a versatile and efficient drug-delivery system in clinical applications [24,25]. Furthermore, in recent years, it has gained remarkable interest in the field of cancer therapy due to its ability to provide an active targeting [26]. In particular, the accumulation of HSA—as well as compounds bound to HSA—in the tumors is attributed to its efficient interaction with the glycoprotein gp60, a receptor expressed on the endothelial cell surface and overexpressed on tumor cells. The binding of HSA to the gp60 receptor leads to the activation of a receptor-mediated endothelial transcytosis process, resulting in the transport and release of the protein—and other plasma constituents—into the tumor interstitium. Then, HSA binds with high-affinity SPARC (secreted protein acidic rich in cysteine), an extracellular matrix glycoprotein highly expressed in various types of tumors, which favors the active accumulation of HSA and drug-loaded HSA within the tumor [27,28].

Herein, we report the design and development of a novel light-responsive multifunctional nanostructure, where tri-modal cancer treatment strategies—PTT, PDT, chemotherapy—luminescent properties and targeting are integrated into the same scaffold. It consists of a spherical-shaped gold core (Au) with a silica shell (SiO_2_) able to physically trap photosensitizing molecules within its matrix.

Due to its remarkable photosensitizing and luminescent properties—as well as absorption features—Rose Bengal (RB) was selected to be loaded into the polysiloxane shell [29,30]. Then, the nanoparticle surface was decorated with HSA biomolecules through a covalent conjugation to confer its targeting abilities and additionally use the protein as a carrier of one of the most effective anticancer drugs in clinical chemotherapy, Doxorubicin (DOX) [31]. Although DOX exhibits a wide range of antitumor properties, its propensity to accumulate in normal tissues, limited cellular uptake, and significant side effects hinder its therapeutic efficacy. Therefore, finding a drug delivery route that can reduce its toxicity, reduce DOX distribution throughout the body, and improve its content in tumor regions has become an important goal in this research field [32].

**Figure 1:**
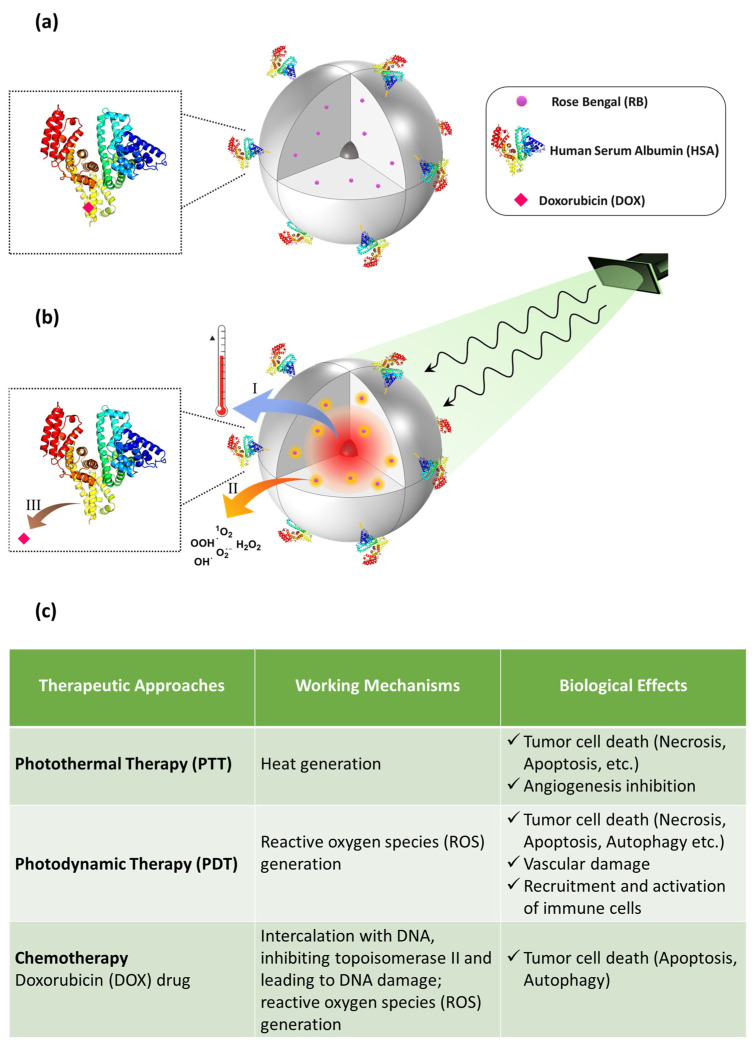
(**a**) Schematic illustration of the implemented nanoplatform (RB-AuSiO_2__HSA-DOX) with a representative example of DOX/HSA interaction and (**b**) light-induced processes: (I) photothermal effects due to irradiation at the plasmon resonance wavelength of the gold core; (II) Rose Bengal molecules activation resulting in the generation of singlet oxygen and other reactive oxygen species; (III) chemotherapy agent release. (**c**) Outline of the therapeutic approaches, working mechanisms and related biological effects addressed with the nanoplatform.

A schematic illustration of the implemented nanoplatform (RB-AuSiO_2__HSA-DOX) is shown in Figure 1. Several approaches regarding strategies to obtain bioconjugated HSA nanoparticles are reported [33]; however, to the best of our knowledge, this is the first example of HSA, covalently bonded to a multifunctional nanoplatform, used as a chemotherapeutic drug carrier and as targeting ligand.

Compared to the nanoplatforms reported to date, RB-AuSiO_2__HSA-DOX aims to exploit the photothermal and photodynamic approaches in combination with the properties of HSA, a natural carrier of drugs in biological fluids, by using a single excitation wavelength to trigger (I) photothermal effects due to irradiation at the plasmon resonance wavelength of gold core; (II) photosensitizers activation resulting in generation of singlet oxygen and other ROS; (III) hyperthermia-enhanced release of the drug. Moreover, the luminescence of the nanosystem could be useful to provide accurate information about its localization in the tumor region, allowing for subsequent efficient treatment.

The obtained nanostructure was fully characterized through transmission electron microscopy (TEM), dynamic light scattering (DLS) and UV-visible spectroscopy. The interaction between DOX-HSA anchored on the nanoplatform was investigated by fluorescence spectroscopy and compared to that of DOX-HSA. Finally, the internalization process of DOX and RB-AuSiO_2__HSA-DOX in MDA-MB-231 breast cancer cells was assessed by confocal imaging.

## 2. Results and Discussion

### 2.1. Design, Synthesis and Characterization of the Multifunctional Nanoplatform

The preparation of gold-core/silica-shell nanoparticles with a surface functionalized with terminal –COOH groups (RB-AuSiO_2__COOH) was performed as previously reported [5], using RB as a photosensitizer and luminescent probe embedded in the silica matrix. In particular, the reverse microemulsion method was used as a synthetic approach since, compared to other common bottom-up strategies [34,35], it ensures the preparation of highly monodispersed particles with finely controlled sizes. Then, the free –COOH groups were activated through EDC/NHS chemistry [36] to react with the N-terminal α-amino groups of HSA biomolecules to yield protein-conjugated nanosystems (RB-AuSiO_2__HSA) through the formation of amide bonds (−CO–NH−).

**Figure 2:**
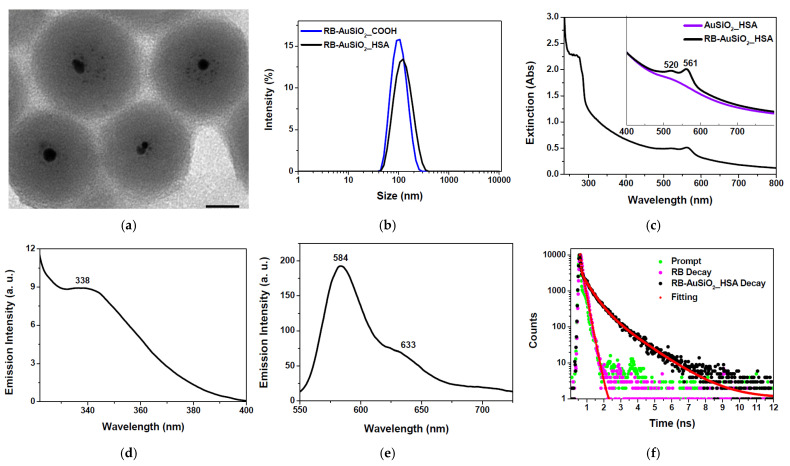
(**a**) Representative TEM image of RB-AuSiO_2__HSA, scale bar = 20 nm. (**b**) The hydrodynamic diameter of RB-AuSiO_2__COOH versus RB-AuSiO_2__HSA. (**c**) Extinction spectrum of RB-AuSiO_2__HSA in water. The inset displays the extinction spectra of AuSiO_2__HSA versus RB-AuSiO_2__HSA in the range 400–800 nm. (**d**) Emission spectrum of RB-AuSiO_2__HSA in water upon excitation at 295 nm. (**e**) The emission spectrum of RB-AuSiO_2__HSA in water upon excitation at 520 nm. (**f**) Time-resolved fluorescence decay of RB versus RB-AuSiO_2__HSA in water upon excitation at 485 nm.

TEM images of the obtained nanoplatforms (Figure 2a) display spherical nanoparticles with an average diameter of 59.33 ± 4.85 nm and a gold core of 6.13 ± 0.88 nm.

Figure 2b shows DLS measurements of RB-AuSiO_2__HSA versus RB-AuSiO_2__COOH. The presence of the protein on the nanoparticle surface produces a significant increase in the hydrodynamic diameter of the bioconjugated nanoparticles compared to the bare bones, with values of 130.57 ± 7.05 nm (PDI = 0.241) and 93.07 ± 2.36 nm (PDI = 0.153), respectively. The obtained results are consistent with reported values, where—after nanoparticle conjugation with HSA—a similar increase in hydrodynamic diameter was observed [37]. Both RB-AuSiO_2__COOH and RB-AuSiO_2__HSA dispersed in water are characterized by negative ζ-potential values, respectively, of −31.48 ± 0.95 and −37.16 ± 0.52 mV. The higher negative charge of the latter confirms the presence of the protein on the nanoparticle surface [23].

The extinction spectrum of RB-AuSiO_2__HSA in water displays the typical features of the nanoplatform components (Figure 2c), i.e., HSA, RB and gold core. In particular, an intense peak—mainly attributed to HSA and RB molecules—is observed in the 250–280 nm range, whereas at lower energy, a broad band between 450 and 600 nm—corresponding to the surface plasmon resonance of gold and to the characteristic absorption peaks of the photosensitizer molecules—is observed. In order to highlight the different contributions, the extinction spectra of RB-AuSiO_2__HSA versus AuSiO_2__HSA, gold-silica nanoparticles without encapsulated RB, are reported in the inset. At around 520 nm, the plasmon resonance peak is observed as a shoulder in the spectrum of AuSiO_2__HSA, whereas in that of RB-AuSiO_2__HSA, this spectral feature overlaps with an RB absorption maximum. In fact, compared with the RB in an aqueous solution (Figure S1), which displays absorption maxima at 511 and 548 nm, the molecule physically trapped within the silica matrix exhibits spectral red-shifts, with absorption maxima at 520 and 561 nm, confirming the strong dependence of the RB photophysical properties on the molecular environment [38,39]. Upon irradiation, the RB-AuSiO_2__HSA nanoplatform shows a double emission at 338 nm (Figure 2d) and in the 550–750 nm range (Figure 2e), exciting respectively on the energy levels of the protein (λ_ex_ = 295 nm) or RB (λ_ex_ = 520 nm).

As regards the emission attributed to HSA (Figure 2d), a blue-shift of approximately 6 nm is observed compared to the free protein (Figure S2), due to the greater rigid environment experienced by the biomolecule bound to the nanoparticle surface, whereas, as regards the emission of the RB (Figure 2e), a red-shift is observed in agreement with the red-shift observed in absorption.

Time-resolved fluorescence decay measurements were carried out on RB and RB-AuSiO_2__HSA (Figure 2f). Although for the bare RB in solution a biexponential kinetics (τ_1_ = 80 ps, α_1_ = 36.16%; τ_2_ = 182 ps, α_2_ = 63.84%; Chi^2^ = 1.039) was observed, in the case of the RB within the nanoplatform a tri-exponential decay (τ_1_ = 47 ps, α_1_ = 17.93%; τ_2_ = 484 ps, α_2_ = 54.08%; τ_3_ = 1.36 ns, α_3_ = 27.99%; Chi^2^ = 1.265) was recorded. The estimation of the corresponding average lifetimes (RB = 145 ps; RB-AuSiO_2__HSA = 274 ps) reveals a higher value for RB-AuSiO_2__HSA than that obtained for RB in solution due to the greater rigid environment experienced by RB molecules when encapsulated into the polysiloxane matrix.

**Figure 3:**
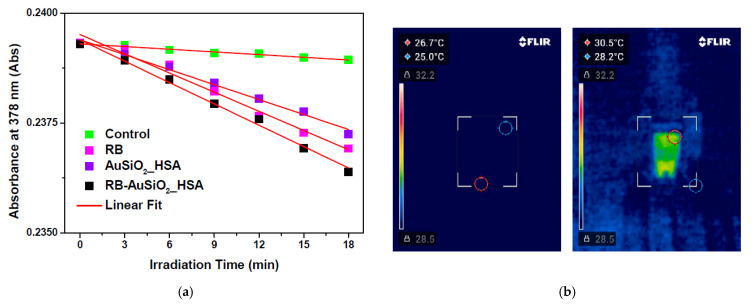
(**a**) Plotting of ABDA absorption at different irradiation times (0–18 min) in water (control) and in the presence of RB, AuSiO_2__HSA and RB-AuSiO_2__HSA. (**b**) Infrared thermal images of (**left**) water (control) and (**right**) RB-AuSiO_2__HSA nanoparticles dispersed in water (light-exposure 520 nm, 90 min).

In order to evaluate the photo-induced properties of the RB-AuSiO_2__HSA nanoplatform, singlet oxygen generation and photothermal effects were assessed. In particular, the singlet oxygen generation capability was evaluated by a chemical method using 9,10-Anthracenediyl-bis(methylene)dimalonic acid (ABDA) as detection probe [40]. The photooxidation of ABDA in the presence of AuSiO_2__HSA, RB and RB-AuSiO_2__HSA was monitored by measuring its absorbance at 378 nm. As shown in Figure 3a, while the absorbance attenuation of the probe was negligible for the control solution, its absorption intensity gradually decreased upon increasing irradiation time in the presence of AuSiO_2__HSA. As reported, the photoexcitation of metal nanoparticles at the surface plasmon resonance bands results in singlet oxygen and ROS production [41,42]. A higher production of singlet oxygen was observed in the presence of RB, as highlighted by the increased time-dependent photobleaching slope. Finally, in the case of the RB-AuSiO_2__HSA nanoplatform, the singlet oxygen generation results are greater than that produced in the case of the bare RB as well as of gold-silica nanoparticles without RB.

According to the previously reported method [15], singlet oxygen generation was quantitatively estimated by calculating the total number of moles produced upon photoirradiation at 520 nm for 18 min, obtaining values of 4.07 × 10^−10^ for AuSiO_2__HSA, 4.82 × 10^−10^ for RB and 6.00 × 10^−10^ moles for RB-AuSiO_2__HSA.

Then, the heat generation of the nanoplatform under continuous irradiation at 520 nm was investigated. As clearly demonstrated by thermal images acquired after light exposure of 90 min (Figure 3b), photothermal heating of the RB-AuSiO_2__HSA dispersion and the surrounding environment was highlighted, whereas a non-significant temperature variation was observed for the control solution. As expected, the temperature increase was relatively small, about 4 °C, due to the small diameter of the gold core and the low power of the light source used, but it was significant to demonstrate the feasibility of the nanosystem.

### 2.2. Doxorubicin Binding to HSA and HSA-Conjugated AuNPs

Fluorescence quenching is considered a useful method for measuring drug-protein binding affinities [43,44,45]. We studied the interactions of DOX with HSA, using the quenching of the tryptophan fluorescence (Trp-214) as a probing tool, investigating the fluorescence behavior of the protein-free in solution and bound on the surface of the nanoparticles in the presence of increasing concentrations of the drug.

**Figure 4:**
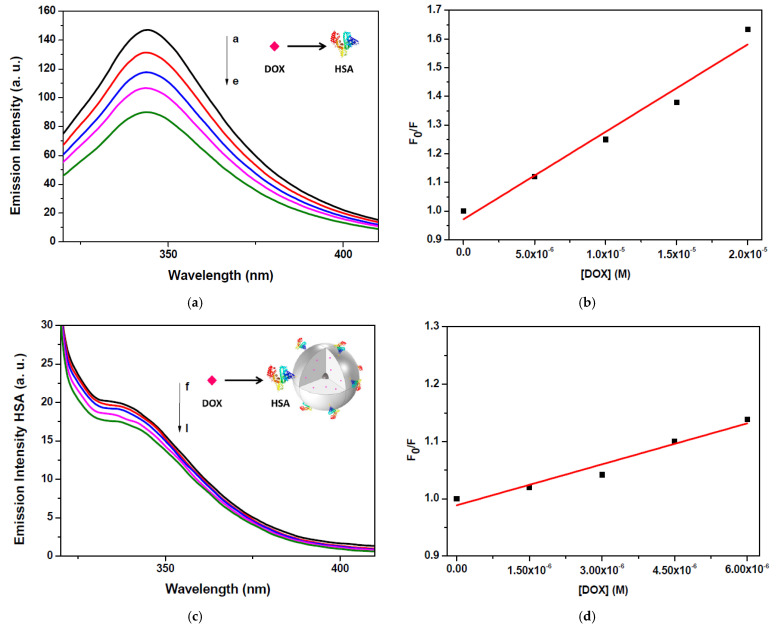
(**a**) Fluorescence emission spectra of HSA (10 μM) in the presence of increasing concentrations of DOX (a–e). (**b**) Stern-Volmer plot for the interaction of DOX with HSA. The molar concentration of DOX in the samples a–e was 0, 5, 10, 15 and 20 μM; λ_ex_ = 295 nm. (**c**) Fluorescence emission spectra of RB-AuSiO_2__HSA nanoparticles (1.5 μM) in the presence of increasing concentrations of DOX (f–l). (**d**) Stern-Volmer plot for the interaction of DOX with HSA-coated nanoparticles. The molar concentration of DOX in the samples f–l was 0, 1.5, 3, 4.5 and 6 μM; λ_ex_ = 295 nm.

As shown in Figure 4a, in the presence of DOX, the luminescence intensity of the protein decreases gradually as the concentration of the ligand increases, reaching a 20% reduction in the equimolar condition. Then, in order to investigate the quenching mechanism induced by DOX, the fluorescence data were analyzed according to the Stern-Volmer equation:

(1) $$\frac{F_{0}}{F}=1+k_{q}\tau_{0}\left[Q\right]$$

where *F_0_* and *F* are the HSA fluorescence intensities in the absence and presence of quencher (i.e., DOX), respectively, k*_q_* is the bimolecular quenching constant, τ_0_ the average lifetime of the protein without quencher (5.71 ns) [44] and [*Q*] is the concentration of quencher. The Stern-Volmer plot reported in Figure 4b displays a good linear relationship, suggesting the occurrence of a single quenching mechanism, with a rate constant of 5.45 × 10^12^ M^−1^ s^−1^, in agreement with the value already reported for DOX/HSA interactions [46]. Since this value is much higher than the maximum value for diffusion-controlled quenching (2.0 × 10^10^ M^−1^ s^−1^), a static quenching mechanism occurs [47]. A noticeable change was observed when DOX binds to HSA-coated nanoparticles compared to its binding to a HSA-free solution. In particular, as shown in Figure 4c, in the presence of DOX, the luminescence intensity of the protein decreases gradually as the concentration of the drug increases, reaching a 2% reduction in the equimolar condition versus a 20% reduction observed in the case of the free protein. This could be attributable to a lower accessibility of the drug molecules to the binding sites of the HSA anchored to the nanostructure compared to those of the free one. The quenching rate constant for DOX/HSA nanoparticles derived from the Stern-Volmer plot (Figure 4d) is 4.05 × 10^12^ M^−1^ s^−1^, a value lower than that obtained for the DOX/HSA interaction.

### 2.3. Confocal Analysis of DOX and RB-AuSiO_2__HSA-DOX in MDA-MB-231 Cells

**Figure 5:**
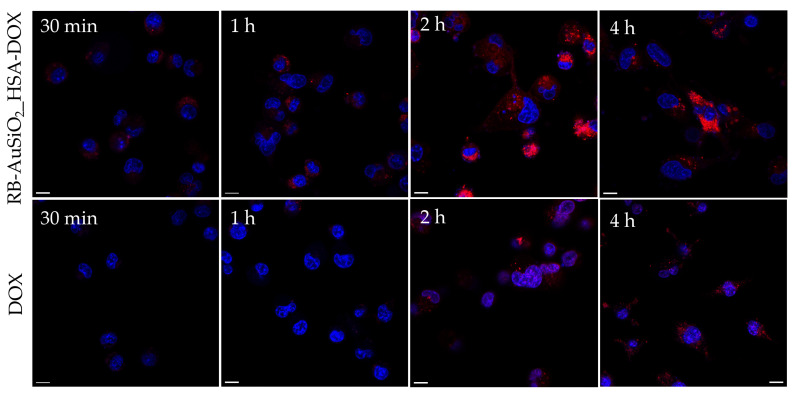
Representative confocal microscopy imaging of MDA-MB-231 cells in the presence of RB-AuSiO_2__HSA-DOX and DOX (red signal). Nucleus were stained with Hoechst (blue signal). Scale bar: 10 µm.

To observe the behavior of RB-AuSiO_2__HSA-DOX and to allow the comparison with DOX, MDA-MB-231 breast cancer cells were incubated with the HSA-coated nanoparticles or the free DOX both at 1 µM of DOX and observed under confocal microscopy imaging at different time points (Figure 5). Free DOX appeared in the nucleus of the cells 2 h after incubation and could also be distinguished in the cytoplasm at longer incubation time. However, the fluorescence of the compound was weak, indicating a slow internalization process. On the contrary, RB-AuSiO_2__HSA-DOX was detectable 30 min after incubation in the cell cytoplasm, and then the accumulation of the nanoparticle increased with time. Once the absence of DOX in the nucleus of the cells in the nanoparticle’s formulation is noticed. We can, therefore, observe an efficient cell internalization of the HSA-coated nanoparticles with effective transport of DOX within the cell. Unlike free DOX, nanoparticles containing DOX were clustered in cytoplasmic vesicles.

The main mechanisms of action of DOX are (i) the intercalation into DNA and disruption of topoisomerase-II-mediated DNA repair and (ii) the generation of free radicals and their damage to cellular membranes. Therefore, DOX needs to reach the nucleus of the cells to efficiently kill cancer cells.

Therefore, in the absence of external stimuli—such as exposure to irradiation and consequent thermal release—the chemotherapeutic drug remains bound to the nanoplatform, thus unable to exhibit its cytotoxic action.

## 3. Materials and Methods

### 3.1. Materials

4-(1,1,3,3-Tetramethylbutyl)phenyl-polyethylene glycol (Triton X-100), hydrogen tetrachloroaurate (III) trihydrate (HAuCl_4_·3H_2_O), sodium 2-mercaptoethanesulfonate (MES), sodium borohydride (NaBH_4_), 4,5,6,7-tetrachloro-2′,4′,5′,7′-tetraiodofluorescein disodium salt (Rose Bengal, RB), (3-aminopropyl)triethoxysilane (APTES), tetraethoxysilane (TEOS), ammonium hydroxide solution (28% *w*/*w*), N-(3-dimethylaminopropyl)-N′-ethylcarbodiimide hydrochloride (EDC), N-hydroxysuccinimide (NHS), human serum albumin (HSA), doxorubicin hydrochloride (DOX) and phosphate buffer saline tablets were purchased from Sigma-Aldrich (Saint Louis, MO, USA). 11-Triethoxysilylundecanoic acid (95%) and N-(3-triethoxysilyl) propylsuccinic anhydride (94%) were purchased from ABCR (Karlsruhe, Germany).

Ultrapure water (Milli-Q, 18 MΩ·cm) was used for the preparation of the aqueous solutions and for all rinses. Phosphate buffer solution (PBS, pH 7.4) was prepared by dissolving one phosphate buffer saline table in 200 mL of Milli-Q water. All other solvents used (n-hexanol, cyclohexane, isopropanol and dimethyl sulfoxide) were of analytical grade.

### 3.2. Synthesis of Nanoparticles

A water-in-oil (W/O) microemulsion was prepared by mixing 3.6 mL of Triton X-100, 3.6 mL of n-hexanol, 15 mL of cyclohexane and a water solution consisting of 0.9 mL HAuCl_4_·3H_2_O (12.75 mM), 0.9 mL MES (36.5 mM) and 0.3 mL NaBH_4_ (423 mM), followed by the addition of RB (2 mg/0.1 mL water), 0.010 mL of APTES and 0.150 mL of TEOS. After 30 min, 0.080 mL of ammonium hydroxide solution was added. The mixture was stirred overnight at room temperature. Then, the functionalization of the nanoparticle surface with carboxyl-terminated aliphatic chains was performed by adding 0.015 mL of 11-triethoxysilylundecanoic acid after 24, 24 + 3, and 48 h. Lastly, the silane coupling agent N-(3-triethoxysilyl) propylsuccinic anhydride (0.015 mL) was added after 48 + 3 h to improve the colloidal stability. The mixture underwent overnight stirring at room temperature. Subsequently, the breakdown of the microemulsion was induced by the addition of isopropanol and water in a volume ratio of 1:1:1. Purification steps employing centrifugal ultrafiltration (Vivaspin 20 PES, 100,000 MWCO, Sartorius, Gottingen, Germany) ensured the complete removal of all unreacted species. The resulting nanoparticles (RB-AuSiO_2__COOH) were ultimately dispersed in water to a final volume of 20 mL.

The same protocol described so far, without the addition of RB, was followed to obtain AuSiO_2__COOH nanoparticles.

Both AuSiO_2__COOH and RB-AuSiO_2__COOH nanoplatforms were conjugated with HSA using EDC-NHS chemistry, which results in the formation of a covalent bond between the carboxyl group (−COOH) of the nanoparticle surface coating agent and the N-terminal group of the protein. 1 mL of EDC (26 mM) and 1 mL of NHS (24.3 mM) were mixed under stirring to 1 mL of nanoparticles solution. After 50 min, 3 mL of PBS was added, followed by the addition of 1 mL of an aqueous solution of HSA (2.77 μM). The mixture was stirred overnight at room temperature.

Finally, the nanostructures were subjected to purification cycles through centrifugal filter devices (Vivaspin 20 PES, 100,000 MWCO, Sartorius, Gottingen, Germany) to eliminate unconjugated protein and unbound reaction components. In particular, the presence of unconjugated protein in the cleaning water was monitored by fluorescence spectroscopy and further washing processes were performed until no protein fluorescence was detected. The resulting solution was concentrated to a final volume of 1 mL. The amount of protein conjugated to nanoparticles was estimated based on HSA emission, according to the calibration curve of the emission intensity versus the concentration of HSA in water solution (Figure S3).

### 3.3. Characterization of Nanoparticles

The synthesized AuSiO_2__HSA and RB-AuSiO_2__HSA nanoparticles were characterized by transmission electron microscopy (TEM), dynamic light scattering (DLS) and UV-vis spectroscopy.

The morphology was examined utilizing a JEOL 2010F transmission electron microscope (Tokyo, Japan). The samples were prepared by applying a diluted colloidal solution onto 200 mesh carbon-coated copper grids. Following the solvent’s evaporation at room temperature, the nanoparticles were observed at an operating voltage of 80 kV.

The average diameter of nanoparticles was determined by TEM analysis; one hundred particles were measured to determine the average particle size. Data were presented as mean values ± standard deviation, and Origin software 9.6.5 was used to process them.

The average diameter measured by TEM was used to calculate the concentration of the nanoparticle samples, according to Equation (2) [48].

(2) $$C=\frac{N_{Total}}{NVN_{A}}$$

where *N_Total_* is the total number of gold atoms added to the reaction solution, *N* is the average number of gold atoms per nanosphere (*N* = 30.90 d^3^, with d = nanosphere diameter in nm), *V* is the volume of the reaction solution in liter, and *N_A_* is the Avogadro’s constant.

Hydrodynamic size and ζ-potential values were determined using a Zetasizer Nano ZS instrument from Malvern (Malvern, UK) (632.8 nm, 4 mW HeNe gas laser, avalanche photodiode detector, 173° detection). Glass cuvettes (1 cm × 1 cm) and disposable folded capillary zeta cells were employed for the measurements and the results expressed as average of three measurements. Origin software was used to process the data.

Extinction and excitation/emission spectra were acquired using a PerkinElmer Lambda 900 spectrophotometer and a Perkin-Elmer LS-50B spectrofluorometer (Waltham, MA, USA). Time-resolved photoluminescence measurements were performed by means of an Edinburgh Instruments’ FLS 1000 fluorometer (Livingston, UK) using a picosecond pulsed diode laser at 485 nm as the excitation source. Quartz cuvettes with a 1 cm × 0.4 cm light path were used for the measurements.

Average lifetimes were calculated according to the formula (Equation (3)):

(3) $$Average\;lifetime=\tau_{1}\alpha_{1}+\tau_{2}\alpha_{2}+\tau_{3}\alpha_{3}\ldots$$

where *τ_n_* are the measured lifetimes and *α_n_* the relative amplitudes.

The singlet oxygen generation by RB, AuSiO_2__HSA, as well RB-AuSiO_2__HSA nanoparticles was investigated by monitoring the chemical oxidation of the molecular probe ABDA (Sigma-Aldrich, St. Louis, MO, USA) in aqueous solution. A volume of 15 µL of ABDA solution (2 mg/mL DMSO) was mixed with 715 μL of RB or nanoparticle solutions and placed in a quartz cuvette with an optical path length of 0.2 cm. The absorption spectra were recorded upon irradiation at 520 nm for 18 min (six cycles of 3 min each) using a Xenon discharge lamp (Hamamatsu, Shizuoka, Japan), which delivered an equivalent power of 20 kW for 8 µs. For comparative purposes, a similar ABDA solution was dispersed in water and irradiated under the same conditions.

The photothermal behavior of RB-AuSiO_2__HSA nanoparticles was investigated under a 450 W continuous Xenon lamp irradiation using an experimental setup like the one previously reported [5]. Briefly, an incident light beam (520 nm) impinged on a 1 cm × 0.4 cm quartz cuvette containing 1 mL of nanoparticle solution. A thermal camera (C-3X model, by FLIR), characterized by a sensitivity of 0.07 °C and placed perpendicular to the incident light (forming a 90-degree angle with the surface of the cuvette), was used to monitor the photo-induced temperature variations. The chamber temperature before the start of irradiation for both the control solution (water) and the sample was 25 °C. After 90 min of irradiation, a thermal image showing the value of the minimum and maximum temperature recorded in the area of interest was acquired.

### 3.4. DOX/HSA and DOX/HSA-Conjugated Nanoparticles Interaction Studies

The interaction of DOX with HSA was investigated by observing the intrinsic fluorescence of the protein in the presence of different drug concentrations. Fluorescence measurements were performed at a DOX/HSA molar ratio of 0/1, 0.5/1, 1/1, 1.5/1 and 2/1. Then, the interaction of DOX with HSA-conjugated nanoparticles was investigated using a DOX/HSA molar ratio of 0/1, 1/1, 2/1, 3/1 and 4/1. All samples were prepared in Milli-Q water, and quartz cuvettes with a 1 cm × 0.4 cm light path were used for all measurements.

### 3.5. Cell Culture and Confocal Microscopy

MDA-MB-231 human breast carcinoma cells were cultured in DMEM L-Glut, enriched in 10% FBS. The cells were grown at 37 °C, in the presence of 5% CO_2_, and trypsinized for passage. Cells (50,000) were seeded in Lab-Tek chamber-I one day before the confocal microscopy study to allow cell attachment. Cells were incubated with DOX and RB-AuSiO_2__HSA-DOX, and the nucleus was labeled with Hoechst 33342 (5 µg/mL). PBS was used as a control. Fluorescence microscopy images were acquired using a confocal laser-scanning microscope (LSM 710 Carl Zeiss, Jena, Germany) in the combi mode. A Plan-Apochromat ×63 objective (Nikon, Melville, NY, USA) was used. The fluorescent signal was obtained with a 488 nm excitation laser at 5%, and the fluorescence was recorded from 550 to 650 nm. For the Hoechst signal, excitation was performed at 405 nm (2% intensity), and emission was collected from 450 to 500 nm. Images were processed using Fiji software 2.9.0. The biological experiments were performed twice on more than 50 cells. The experiments were performed at the MicroCell facility (Optical Microscopy-Cell Imaging, A. Grichine and M. Pezet), IAB Grenoble.

## 4. Conclusions

A novel nanoplatform with luminescent, photosensitizing and photothermal capabilities was synthesized and fully characterized. These properties arise from the combination of the molecular properties of RB (luminescent and photosensitizer) with the thermoplastic properties of gold at the nanoscale. To enhance the theranostic performances of these nanosystems, their surface was functionalized with HSA, exploiting it as a carrier of a chemotherapeutic drug. DOX was employed as a model chemotherapeutic drug; it exhibits a wide range of antitumor capabilities but a limited cellular uptake, which hinders its effectiveness in cancer chemotherapy. Then, the DOX interaction with HSA in solution or anchored to the nanoparticle surface was investigated, shedding light on the different accessibility of the drug molecules to the HSA binding sites in the two different conditions.

Combination therapy is usually regulated by two different wavelengths of light or other stimuli, which leads to complex operating procedures and subsequent systemic side effects, which affect the therapeutic efficacy. Herein, we designed a multifunctional light-activated nanoplatform using a single excitation wavelength.

Finally, confocal imaging experiments were performed following the internalization process in MDA-MB-231 breast cancer cells of DOX and DOX carried by the nanosystem. In particular, we have demonstrated that RB-AuSiO_2__HSA-DOX nanoplatforms are internalized into cells, mainly into the cytoplasm, at early time of incubation compared to the free drug.

At a glance, we presented a proof of concept for the design and development of a multifunctional nanosystem as a carrier of an anticancer drug to ensure improved drug internalization. Further studies are currently underway to enhance the photothermal performance of the plasmonic gold core, using shapes with better light-heat conversion capacity than spheres, such as nanostars and nanobipyramids, and to monitor the thermal release of the chemotherapeutic drug from the nanosystem and the consequent cellular localization under different irradiation exposure doses. Then, before moving on to in vivo studies, in-depth biological investigations will be performed to optimize and reveal cell behavior and cancer cell-killing efficacy, exploiting the synergistic combination of photothermal, photodynamic and chemotherapeutic effects of the stimuli-responsive nanoplatforms.

## Data Availability

The original contributions presented in this study are included in the article/Supplementary Materials. Further inquiries can be directed to the corresponding authors.

## Supplementary Materials

The supporting information can be downloaded at: https://www.mdpi.com/article/10.3390/ijms252413701/s1.
