# Supplementary material for: Conjugated Human Serum Albumin/Gold-Silica Nanoparticles as Multifunctional Carrier of a Chemotherapeutic Drug
Source: Int J Mol Sci. 2024 Dec 21;25(24):13701. doi: 10.3390/ijms252413701 (PMC11678608; doi:10.3390/ijms252413701)
Supplement: Supplementary file 1 [file ijms-25-13701-s001.zip › ijms-3367265-supplementary.pdf]

International Journal of
Molecular Sciences

Supplementary Material

Conjugated Human Serum Albumin/Gold-Silica Nanoparticles as multifunctional carrier of a chemotherapeutic drug

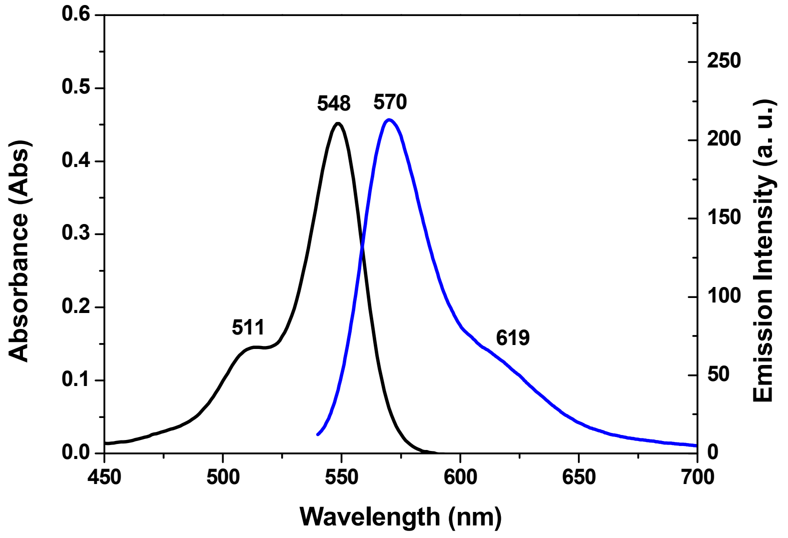

Figure S1. Absorption and emission spectra of RB in aqueous solution (4.12 μM).

(a)

(b)

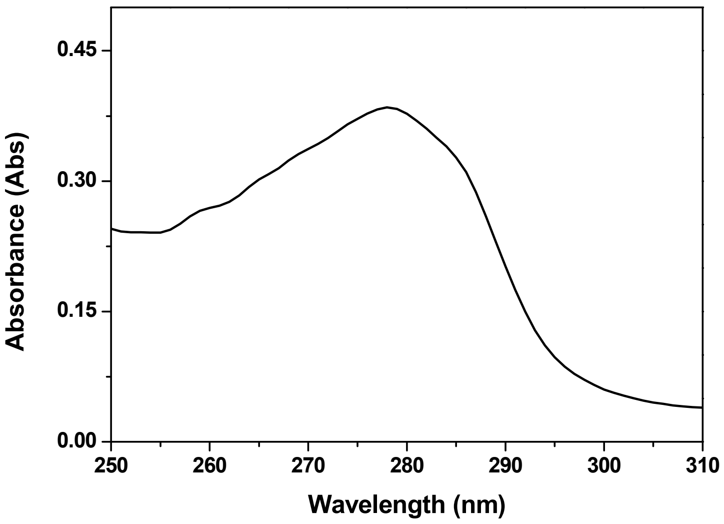

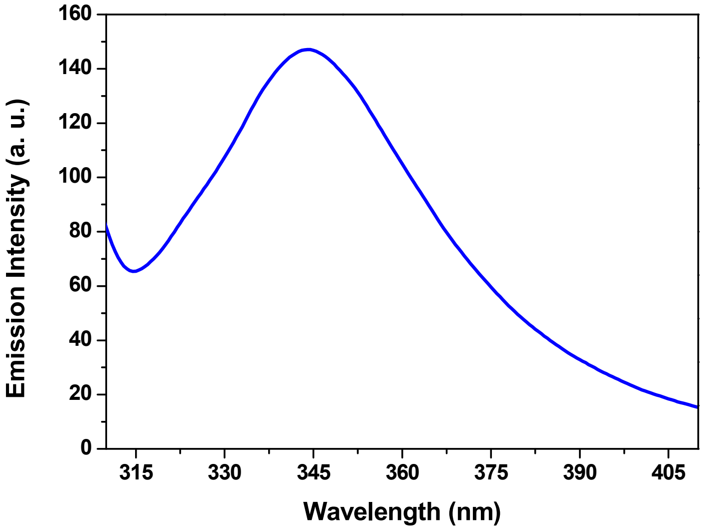

Figure S2. (a) Absorption and (b) emission spectra of HSA in aqueous solution (10 μM).

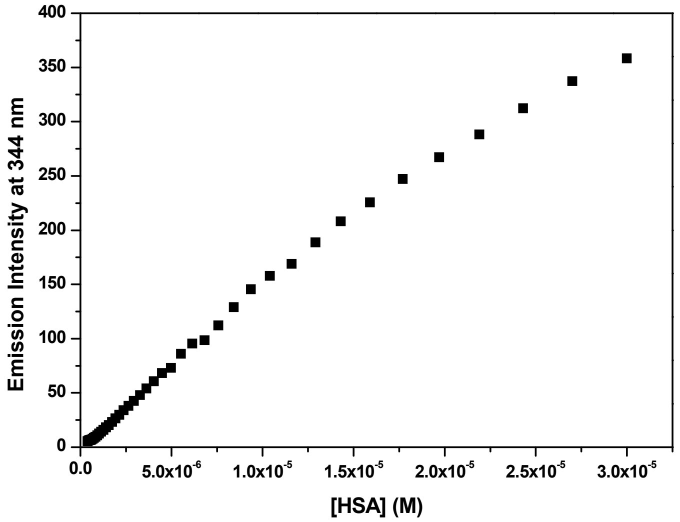

Figure S3. Calibration curve of the emission intensity versus concentration of HSA in water solution.
